# A training goal-oriented categorization model of high-intensity interval training

**DOI:** 10.3389/fphys.2024.1414307

**Published:** 2024-06-18

**Authors:** Thomas L. Stöggl, Tilmann Strepp, Hans-Peter Wiesinger, Nils Haller

**Affiliations:** ^1^ Department of Sport and Exercise Science, Paris Lodron University Salzburg, Salzburg, Austria; ^2^ Red Bull Athlete Performance Center, Thalgau, Austria; ^3^ Institute of Nursing Science and Practice, Paracelsus Medical University, Salzburg, Austria; ^4^ Institute of General Practice, Family Medicine and Preventive Medicine, Paracelsus Medical University, Salzburg, Austria; ^5^ Department of Sports Medicine, Rehabilitation and Disease Prevention, Johannes Gutenberg University, Mainz, Germany

**Keywords:** aerobic and anaerobic energy contribution, endurance performance, neuromuscular, sprint, speed endurance training

## Abstract

There are various categorization models of high-intensity interval training (HIIT) in the literature that need to be more consistent in definition, terminology, and concept completeness. In this review, we present a training goal-oriented categorization model of HIIT, aiming to find the best possible consensus among the various defined types of HIIT. This categorization concludes with six different types of HIIT derived from the literature, based on the interaction of interval duration, interval intensity and interval:recovery ratio. We discuss the science behind the defined types of HIIT and shed light on the possible effects of the various types of HIIT on aerobic, anaerobic, and neuromuscular systems and possible transfer effects into competition performance. We highlight various research gaps, discrepancies in findings and not yet proved know-how based on a lack of randomized controlled training studies, especially in well-trained to elite athlete cohorts. Our HIIT “toolbox” approach is designed to guide goal-oriented training. It is intended to lay the groundwork for future systematic reviews and serves as foundation for meta-analyses.

## 1 Introduction

High-intensity interval training (HIIT) is a well-established training method that has experienced a massive increase in research interest, particularly since the new millennium (Ekkekakis et al., 2023). Various publications have shown that HIIT is a time-efficient training method that elicits cardiorespiratory, metabolic, and skeletal muscle adaptions which ultimately improve sports performance (for review Midgley et al., 2006; Dolci et al., 2020). However, in the majority of reviews on HIIT, it is mentioned that compared with the volume of research that describes the physiological adaptations to endurance exercise training in sedentary and recreationally trained individuals, little work has examined the physiological and performance responses of competitive or highly trained athletes (Billat, 2001a; Laursen and Jenkins, 2002; Midgley et al., 2007).

In addition, in the various original articles or reviews, only the effects of selected types of HIIT on single variables linked to key performance indicators of endurance capacity, e.g., aerobic capacity (VO_2max_), anaerobic capacity, maximal speed or repeated-sprint ability (RSA) were analyzed. A more holistic view of the facets of endurance performance is missing. Furthermore, there is inconsistency across publications on how HIIT is defined, termed, and categorized (Tschakert and Hofmann, 2013; Gibala et al., 2014; Weston et al., 2014; Rosenblat et al., 2020; Boullosa et al., 2022; Coates et al., 2023). In the majority of publications, interval training is defined by repeated short to long (10-s to 5-min) bouts of rather high intensity (Billat, 2001a) interspersed by periods of rest or low intensity to allow partial but often not full recovery (Laursen and Jenkins, 2002; Gibala and Jones, 2013) that usually takes less than 30-min to perform (Thompson, 2017). It is evident that this general description of interval training can be considered as a concept rather than a specific definition, as there are many ways to plan an interval session based on this framework.

Over the past decades, researchers and practitioners have developed numerous types of interval training and given them more specific definitions and nomenclatures depending on the characteristics of the training (Daniels and Scardina, 1984). These approaches can be useful in specifying the training goal and targeting the athletes’ weaknesses. However, this has also led to an inflationary creation of interval training types in the literature with inconsistencies and variations across programs, particularly in types such as aerobic, anaerobic (Chamari and Padulo, 2015), short, long (Billat, 2001a; Tschakert and Hofmann, 2013), intermittent (Christensen et al., 1960; Tschakert and Hofmann, 2013), supramaximal (Billat, 2001b; Gibala et al., 2006), all-out intervals (Gibala et al., 2006), sprint interval training (SIT) (Burgomaster et al., 2005; Weston et al., 2014), repeated-sprint training (RST) (Buchheit and Laursen, 2013a), and speed endurance training (SET) (Mohr et al., 2007; Iaia and Bangsbo, 2010). Specifically, there are conflicting terms and definitions related to the description of exercise mode, interval and recovery intensity and duration, recovery activity (i.e., passive, active), number of intervals and sets, or intensity control guidelines (i.e., oxygen uptake, velocity, heart rate, perceived effort, etc.).

Due to the need for more consensus between researchers and practitioners on terminology and definitions, developing a HIIT categorization model is necessary. This model is particularly important to adequately understand and prescribe HIIT to reach specific training goals and to compare and meta-analyze study results. Several author groups have tried to categorize HIIT (Buchheit and Laursen, 2013a; Tschakert and Hofmann, 2013; Weston et al., 2014; Coates et al., 2023), but a comprehensive performance and goal-orientated model, including a wide range of interval training concepts, still needs to be added. Thus, in this review, we propose a categorization model of HIIT, attempting to find the greatest consensus on various definitions in the literature, and present the idea of a “training goal”-oriented and applied HIIT concept.

## 2 Proposed HIIT categorization

**FIGURE 1:**
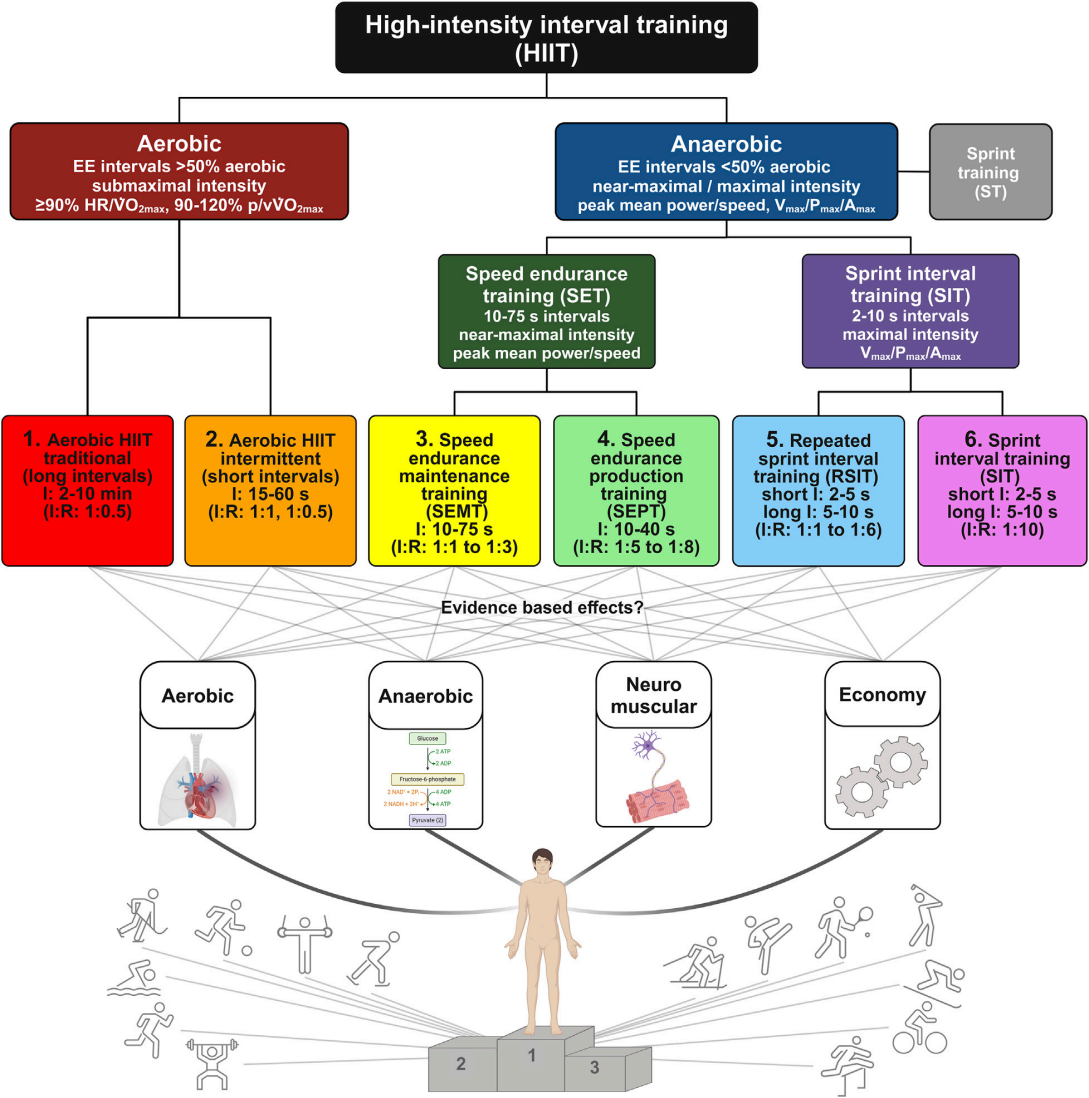
A training goal-oriented categorization model of HIIT. The model distinguishes between aerobic and anaerobic energy contributions and concludes with six different types of HIIT derived from the literature and based on the interaction of interval:recovery ratio. Subsequently, possible effects of the six types of HIIT on important physiological systems, which are required differently depending on the sport, are visualized. The terms represent an overarching categorization of physiological adaptations. “Aerobic” includes aspects such as mitochondrial density, fat reliance, muscle fiber type optimization, aerobic enzyme enhancement. “Anaerobic”, e.g., up/down glycolytic enzymes, lactate dehydrogenase isoenzyme, monocarboxylate transporter. “Neuromuscular”, e.g., force production, contraction time, neural signaling. “Economy”, i.e., metabolic and mechanical efficiency. The gray box is included in the model for the purpose of completeness but is not discussed in detail in this review. (Created with BioRender.com). HIIT = high-intensity interval training; EE = energy expenditure; HR = heart rate; VO_2max_ = aerobic capacity; p/vVO_2max_ = power/velocity at aerobic capacity; V_max_ = maximal velocity; P_max_ = maximal power; A_max_ = maximal acceleration; ST = sprint training; SET = speed endurance training; SIT = sprint interval training; SEMT = speed endurance maintenance training; SEPT = speed endurance production training; RSIT = repeated sprint interval training; I:R = interval:recovery ratio.

Hierarchically, the categorization model starts with HIIT, the interval-based concept of the generic term “High-intensity training” (HIT). Aware that continuous efforts with “high” intensity (e.g., competitions, time trials, competition simulation; note the definition of “high” depends on the type of HIIT, see further below) and high-intensity strength training (e.g., maximal power output, high loads (Hurley et al., 1984; Mentzer, 1993; Bruhn et al., 2006)) can also be categorized at the same level as HIIT, we focus on the interval-based concepts in this review (Figure 1).

The second level further distinguishes between aerobic and anaerobic energy contribution and incorporates the target intensity within the intervals, as previously mentioned by Rosenblat et al. (2020). Depending on the a) proportion of the aerobic vs anaerobic energy system and b) target intensity for the intervals (e.g., near maximal/maximal/all-out vs submaximal intensity relative to submaximal or maximal anchors (Jamnick et al., 2020)), we differentiate between aerobic and anaerobic HIIT. According to Billat (2001a), aerobic HIIT is defined as interval training where the energy demand during the intervals elicits aerobic metabolism at a higher rate (i.e., ≥50%) than the anaerobic metabolism. Based on various studies focusing on the energy contribution of maximal efforts across various distances/times, the crossover point where aerobic and anaerobic energy contributes equally occurs around 2 min (Åstrand et al., 2003) or 600 m respectively 75 s in running (Duffield et al., 2005; Laursen, 2010), 60–90 s in cycling (Gastin and Lawson, 1994a; b; Craig et al., 1995; Craig and Norton, 2001), and similar durations in swimming (Rodríguez and Mader, 2011). In accordance with Gastin (2001), we have therefore decided to set the cutoff between anaerobic and aerobic HIIT of classical interval-based HIIT at an interval duration of 75 s.

### 2.1 Aerobic HIIT

On the third level, aerobic HIIT can be categorized into several forms (Figure 1). These include traditional or “long” aerobic intervals of 2–10 min, e.g., 4 × 4 min (Helgerud et al., 2007; Sandbakk et al., 2013; Seiler et al., 2013; Rønnestad et al., 2014), and intermittent or “short” aerobic intervals (Christensen et al., 1960; Laursen and Jenkins, 2002; Rønnestad et al., 2015) lasting around 15 s (Helgerud et al., 2007; Dolci et al., 2020) to 60 s, e.g., 30 × 30 s (Gibala et al., 2012; Stöggl et al., 2023). Short aerobic intervals are performed with a “high”, but not maximal target intensity enabling athletes to exercise at intensities at or above competition speed for extended durations (Gullstrand, 1996) while long aerobic intervals are usually performed with slightly lower intensity compared to short aerobic intervals (Rønnestad et al., 2015). Additionally, there are mixed interval structures among practitioners with pyramidal patterns involving increasing, decreasing, or a combination of increasing and decreasing interval durations, such as 1-2-3-4–5 min, 5-4-3-2-1 min, or 1-2-3-4-3-2-1 min intervals (Vaccari et al., 2020). Typical interval:recovery ratios for aerobic HIIT are 1:0.5 or 1:1 (Rozenek et al., 2007).

The following intensity targets are commonly applied for aerobic HIIT: ≥90% VO_2max_ or ≥90% maximal heart rate (HR_max_) but not maximal intensity (Laursen and Jenkins, 2002; Buchheit and Laursen, 2013a; Buchheit and Laursen, 2013b; Weston et al., 2014); 90%–120% power output/velocity at VO_2max_ (p/vVO_2max_), i.e., maximal aerobic power/speed (MAP/MAS) (Hill and Rowell, 1996; Buchheit and Laursen, 2013a); 70%–110% peak power output (PPO) determined by incremental exercise test (Astorino et al., 2017; Stöggl et al., 2023); power output or speed above critical power/speed (CP/CS) (Jones et al., 2010); rating of perceived exertion (RPE) exceeding 6 on the CR-10-BORG scale (Buchheit and Laursen, 2013a), or ranging between 15–18 (hard—very hard) on the 6 to 20-BORG scale (Buchheit and Laursen, 2013a; Ciolac et al., 2015; Foster et al., 2021; Coates et al., 2023); lactate concentration above the maximal lactate steady state (Tschakert and Hofmann, 2013) or second lactate threshold (Jamnick et al., 2020), greater than 4 mmol/L (Mader, 1976) or even between 7 and 10 mmol/L (Faude et al., 2013; Thum et al., 2017); above the respiratory compensation point/second ventilatory threshold (Meyer et al., 2004).

Besides constant intensity work intervals, “fast start” intervals with subsequent reduced intensity, e.g., 1.5 min at 100% MAP/MAS followed by 3.5 min at 85% MAP/MAS (Rønnestad et al., 2020b), or intervals with varying intensity, e.g., 3 × 30 s at 100% MAP/MAS interspersed with 1 min and a final 1.5 min at 77% MAP/MAS (Bossi et al., 2020), are discussed to be suitable alternatives. As described above, constant-intensity work intervals with lower intensity levels, e.g., <90% HR_max_, will not be categorized as HIIT in the current categorization but would need another interval training terminology, e.g., moderate-intensity interval training (MIIT). Consequently, in the context of performance, “vigorous intensity”, defined by the American College of Sports Medicine as 77%–95% of HR_max_, cannot necessarily be considered as high intensity although it is often excepted as such in the health context (Liguori et al., 2022; Coates et al., 2023). For instance, the 8–12 × 1 min protocol at ∼100% VO_2peak_ performed with healthy men (Little, 2012) can be considered as aerobic HIIT intermittent, whereas the 10 × 1 min intervals at 80%–90% HR_max_ with obese men (Poon et al., 2020) would not fit our model but MIIT.

To reduce complexity and apply types of HIIT that are feasible during training using common sensor technology (e.g., heart rate monitors, power meters), we suggest using the reference levels >90% HR_max_ (VO_2max_) or 90%–120% p/vVO_2max_ for aerobic HIIT. It is important to recognize that these intensity guidelines apply to well-trained individuals; for patients, older or inactive individuals (Coates et al., 2023), adjustments may need to be made due to factors such as reduced endurance capacity.

### 2.2 Anaerobic HIIT

For anaerobic HIIT, the third level is categorized based on the interval duration (i.e., 2–10 s and >10–75 s), interval intensity (see discussion below) and the recovery time between intervals (Figure 1).

#### 2.2.1 Sprint interval training (SIT)

In the context of “anaerobic HIIT” the term “sprint” is often used. However, there is a wide range in describing a sprint leading to difficulties in differentiating between HIIT concepts. For instance, in various publications, the term “sprint” is used for interval or effort durations of 20 s or longer (Bogdanis et al., 1995; Bogdanis et al., 1996; Ball et al., 1999; Nilsson et al., 2004; Burgomaster et al., 2006; Gibala et al., 2006; Iaia et al., 2008; Buchheit and Laursen, 2013a; Vandbakk et al., 2017; Dolci et al., 2020) or intensities that also include intensities below maximum (Weston et al., 2014) or “near-maximum” (Coates et al., 2023). However, a “sprint” is conventionally associated with moving as fast as possible, i.e., reaching maximal velocity (V_max_) or maximal power (P_max_), e.g., measured during an all-out Wingate test, or maximal acceleration/explosiveness from the start (A_max_) (Tabata, 2019).

Within a 100 m maximal running sprint, V_max_ is reached between 60 and 70 m in elite sprinters with running speed already decreasing after 70 m (Majumdar and Robergs, 2011). In contrast, team sports athletes reach their V_max_ between 30 and 40 m (Duthie et al., 2006; Young et al., 2008) and male sport students after 40 m (Babić et al., 2011). For longer interval durations (e.g., >10 s) maximal performance (e.g., reaching V_max_/P_max_/A_max_) is no longer achieved in many cases, as shown in the comparison of 100 m and 200 m world record sprints (Girard et al., 2011), possibly due to pacing strategies (Tibshirani, 1997; Abbiss and Laursen, 2008). Therefore, if the training goal targets are improvement in V_max_/P_max_/A_max_, we hypothesize that 100% V_max_/P_max_/A_max_ or even supramaximal intensities should be reached during HIIT (Bishop et al., 2011; Haugen et al., 2014; Petrakos et al., 2016; Rumpf et al., 2016; Verheijen, 2016).

While successful sprint coaches have also prescribed submaximal “sprints”, i.e., 90%–95% V_max_, over decades (Haugen et al., 2019), a study in soccer players performing sprints at 90% V_max_ once a week over 2 months found no sufficient improvement in performance when compared to a control group (Haugen et al., 2014). In accordance with Girard et al. (2011) and Iaia and Bangsbo (2010), we therefore define SIT as repeated maximal bursts lasting 2–10 s with maximal possible intensity from standstill or using flying sprints, if A_max_ is not the training goal.

According to the recovery duration between single sprint intervals, we differentiate between SIT and repeated SIT (RSIT). SIT uses a 1:10 (Iaia and Bangsbo, 2010) interval:recovery ratio with recovery periods long enough to allow near complete restoration of sprint performance (Balsom et al., 1992; Bishop and Claudius, 2005; Duffield et al., 2009; Verheijen, 2016). From an energetic perspective, it needs to be mentioned, that phosphocreatine (PCr) resynthesis half-life is about 170 s (Hirvonen et al., 1987) with approximately full restoration after 4 min (Spriet et al., 1989). Sufficient recovery time for PCr store resynthesis allows to achieve V_max_/P_max_/A_max_. To comply with the above recommendations, recovery durations of 60–300 s are proposed for SIT (Girard et al., 2011). In contrast, RSIT uses a 1:1 to 1:6 ratio with shorter recovery periods of 10 s (Verheijen, 2016) to maximal 60 s (Spencer et al., 2005). RSIT aims to target the maintenance of V_max_/P_max_/A_max_, with ongoing fatigue (Bishop et al., 2011; Girard et al., 2011). Of note, in another attempt to categorize the HIIT term (Tschakert and Hofmann, 2013), SIT and RSIT were categorized contrary to the concepts above; hence, long recovery for RSIT and short recovery for SIT. Based on the training-goal orientation of our concept we, therefore, follow the definition used in the majority of publications and applications in practice.

Within the context of SIT and RSIT, a further differentiation is possible: a) if the training target is V_max_ or P_max_, flying sprints from a rolling start of 5–10 s (SIT/RSIT long) including the run-up are recommended and b) if the training target is to improve A_max_ shorter intervals of e.g., 2–5 s, starting from standstill, seem sufficient (SIT/RSIT short) (Haugen et al., 2019). To add, that within this article we focus on training concepts related to sports with endurance component. For instance, a pure sprinter will also aim on improving her or his V_max_/P_max_/A_max_ etc. but with distinctly longer recovery periods (e.g., 1–2 min recovery for every second at V_max_ (Haugen et al., 2019) or 1-min recovery for every 10 m sprint (Kadlec and Groeger, 2020)) in total having a minor endurance training component. While this classic “sprint training” (ST) can certainly be categorized as HIIT, it does not fall within the scope of the current HIIT categorization (Figure 1).

#### 2.2.2 Speed endurance training (SET)

Longer anaerobic intervals (>10 s; conventionally defined with ∼30-s) executed as all-out or near-maximum (e.g., 130% vVO_2max_ (Mohr et al., 2007), 93% of speed achieved in a 30-s all-out sprint run (Iaia et al., 2009), 130%–150% PPO (Astorino et al., 2017)) efforts are termed speed endurance training (SET) (Mohr et al., 2007; Iaia and Bangsbo, 2010). To maintain terminological precision, we recommend categorizing “sprints” with >10–45 s interval duration referred to by others as SIT (Gibala et al., 2006; Buchheit and Laursen, 2013a; Sloth et al., 2013; Gist et al., 2014; Coates et al., 2023) to be categorized as SET. In accordance with the differentiation into SIT and RSIT as mentioned above, SET can be further differentiated based on the recovery duration between the intervals (Figure 1). Speed endurance production training (note, for reasons of consistency, we have added a T for “training” to the terminology, hence “SEPT” instead of SEP) is characterized by intervals of 10–40 s (Iaia and Bangsbo, 2010) with highest possible mean power output/velocity (70%–100% V_max_) and long recovery periods (4-6 times the interval duration (Iaia and Bangsbo, 2010; Iaia et al., 2015)) to allow for repeated intervals at the same quality, e.g., 40 s at 125% PPO with 260 s break (Stöggl et al., 2023). Conversely, speed endurance maintenance training (SEMT) employs interval durations of up to 90 s (Iaia and Bangsbo, 2010) (75 s in our model) with shorter recovery periods of 1–3 times the interval duration, e.g., 40 s passive recovery (Iaia et al., 2015) to stress the repeatability with accumulated fatigue. As can be seen here, there is still a wide range of advocated interval durations, exercise intensities (all-out, supramaximal, %V_max_), and recovery durations in SEMT, which complicates a clear-cut training prescription.

We are aware that this distinct categorization into the various types of HIIT is sometimes ambiguous. For instance, the contribution of the aerobic energy system increases from sprint to sprint (e.g., RSIT) and might reach a contribution higher as 50% in later sprints (Gaitanos et al., 1993). In addition, some specific HIIT concepts do not fit easily into any of the categories. For instance, so-called “Tabata training” is defined as training at the intensity that exhausts athletes during the seventh or eighth set of 20-s bicycle exercise bouts with 10-s recovery in between (Tabata et al., 1996). If the 20-s intervals are performed “all-out”, Tabata can be categorized as SEMT according to our proposed categorization. If the intensity of 170% VO_2max_ is “submaximal,” this workout can also be categorized as intermittent aerobic HIIT. Therefore, the chosen target intensity can assist in the assignment into the various HIIT categories. For example, 10 × 1 min with 30 s recovery can be categorized as SEMT if the intensity for the 1-min interval is “maximal mean power” or “as fast as possible”. In contrast, it can be categorized as aerobic HIIT with short (intermittent) intervals if the intensity target is 90%–95% HR_max_/VO_2max_ across the 10 × 1 min at high but not maximal intensity.

## 3 Purpose of the science-based HIIT categorization

The idea of this review was to propose a framework as a fundament for a training goal-oriented HIIT “toolbox”. This should serve as a recommendation which type of HIIT should be applied to develop various pillars of endurance performance, e.g., aerobic, anaerobic, neuromuscular, and economy depending on the needs of the sport and potentials of athletes (Figure 1). To achieve that, more detailed information about the customization of HIIT (type, interval durations, recovery durations, recovery between sessions, etc.) to maximize the various performance related factors in athletes should be provided.

The paradigm underlying training adaptation is that specific training induces increased expressions of proteins with specific physiological functions in skeletal muscles recruited by training (Tabata, 2019). One of our hypotheses is therefore, that HIIT modalities that place the greatest stress on the various cardiorespiratory, metabolic, neuromuscular, and musculoskeletal systems will result in the greatest adaptations in each of these mediators of performance. For instance, are the aerobic HIIT versions the ones that maximize improvements in aerobic endurance performance based on the idea that this type of HIIT provides the highest demand on the oxidative system, e.g., high relative VO_2_ over long training time? Is HIIT where V_max_/P_max_/A_max_ is targeted, leading to greatest increases in V_max_/P_max_/A_max_ capacities? Are HIIT versions that stress the anaerobic energy system the most lead to the greatest improvements in predominantly anaerobic endurance tasks, e.g., “anaerobic” glycolytic performance as in a 200-m run? Is HIIT with the greatest demand on the PCr stores the one that improves the greatest repeated-sprint performance and PCr store capacity (Verheijen, 2016)?

There is also discussion about whether certain HIIT concepts lead to a wide range of adaptations in the area of aerobic, anaerobic, neuromuscular performance and economy at the same time. For instance, in the example above, the question is if RSIT enhances the RSA more than an aerobic HIIT that potentially increases aerobic capacity and therefore might foster the rate of PCr resynthesis. As another example, the “Tabata protocol” was suggested to stress both the aerobic and anaerobic system maximally proposing that “Tabata training to be the ultimate aerobic and anaerobic training method” (Tabata, 2019). Buchheit and Laursen (2013b) propose, that combined with the neuromuscular load and the musculoskeletal strain it is possible to characterize the response to a HIIT session. To answer these questions, this categorization model should serve as a prerequisite for further systematic analyses of existing study results.

## 4 Science of aerobic HIIT–duration, intensity and time at VO_2max_

ÅStrand et al. (1960) already mentioned in 1960 that long aerobic intervals, i.e., 3-min runs at 90%–92% vVO_2max_, are considered to be the best form of interval training to improve VO_2max_ since all cardiorespiratory parameters are at their maximum. In addition, it was proposed that longer intervals of, e.g., 1–3 min, could lead to a superior training effect on cardiorespiratory function compared with short aerobic intervals (30 s). This hypothesis was also supported by other studies (Demarie et al., 2000; Bacon et al., 2013; Wen et al., 2019). However, studies have also shown superior training adaptations for short (30 s) compared to long (5 min) aerobic intervals (Rønnestad et al., 2015; Rønnestad et al., 2020a) in competitive and elite cyclists and studies with both untrained and trained participants have demonstrated increases in VO_2max_ in training concepts with short (6–30 s) as well as longer (2.5–4 min) aerobic intervals (Helgerud et al., 2007; Astorino et al., 2012; Ouerghi et al., 2014; Astorino et al., 2017). In addition, previous studies have demonstrated that supramaximal intermittent exercise (in this paper termed “aerobic intermittent intervals”) have led to marked improvements in VO_2max_ in healthy males and females (Fox et al., 1975; Lesmes et al., 1978). Furthermore, Hill and Rowell (1997) found that the minimum time it took for VO_2max_ to be reached was 60% of T_max_ (the time an athlete can hold when running with vVO_2max_) in a group of highly trained female middle-distance runners. Therefore, HIIT with intervals performed between 50% and 60% of T_max_, i.e., ∼2.5 min (Laursen et al., 2002), might be optimal for improving endurance performance. Hence, there is some discrepancy with respect to the optimal interval duration, e.g., long vs short intervals.

With respect to training intensity, Fox and colleagues (Fox et al., 1973; Fox and Matthews, 1974; Fox et al., 1975; Fox et al., 1977; Fox, 1979) examined the effects of interval training on the human body’s aerobic energy-releasing system. It was suggested that the improvement of the body’s VO_2max_ after interval training is linearly related to the O_2_ demand during the intervals, indicating exercise intensity to be a key factor for improving of aerobic capacity. In this context, Wenger and Bell (1986) suggested that regardless of the initial fitness level the most effective improvement in cardiorespiratory fitness is induced by training at an intensity corresponding to 90%–100% of VO_2max_. In addition, it is stated that interval training with intensities close to vVO_2max_ may maximize the improvement of VO_2max_ and improve mitochondrial density (Brooks et al., 1996). The rationale for using vVO_2max_ in HIIT program prescription is based on the assumption that further improvements in VO_2max_ in the highly trained athlete will only result from exercise intensities at or above vVO_2max_ (Laursen and Jenkins, 2002). It is suggested that a training program consisting of repeated 1- to 8-min runs at 90%–100% vVO_2max_ is the most effective approach for improving VO_2max_ and performance in middle distance runners (Fox et al., 1975). In addition, intermittent intervals allow athletes to cover substantial distances at a high velocity, thereby maximizing the number of powerful muscle contractions to determine muscular adaptations (Noakes, 2003). Hence, aerobic intermittent intervals at vVO_2max_ or higher not only stimulate the cardiovascular system maximally for a longer duration, but also enable athletes to generate a greater power output. This aspect is an additional argument for distinguishing between intermittent and traditional interval-based aerobic HIIT.

It is believed that an optimal stimulus to elicit both maximal cardiovascular and peripheral adaptations is one where athletes spend several minutes per session at or near VO_2max_ (T@VO_2max_), commonly referred to as their “red zone”, e.g., >90% of their VO_2max_ (Wenger and Bell, 1986; Buchheit and Laursen, 2013a; Mølmen and Ronnestad, 2024). T@VO_2max_ is suggested to be at least 7 min (team sports) or >10 min (long-distance runners) (Buchheit and Laursen, 2013a; Dolci et al., 2020) to elicit relevant cardiopulmonary adaptations. However, these assumptions are thus far based on weak scientific evidence. As stated above, the underlying mechanism in the improvement of VO_2max_ is seen in the significant stress on the aerobic system if working at high relative VO_2_ over a prolonged time (Billat, 2001a). In the context of well-trained endurance athletes, aiming for even higher intensities to reach their individual maximum aerobic performance seems advisable (Midgley et al., 2006). However, few studies focus on comparing T@VO_2max_ of different HIIT protocols in an acute setting (Bossi et al., 2020; Rønnestad et al., 2020b) or during a training intervention (Turnes et al., 2016; Rønnestad et al., 2022).

Turnes et al. (2016) have measured T@VO_2max_ of two groups of recreationally trained cyclists differing in interval intensity (∼130% MAP *versus* 105% CP). The authors found a longer T@VO_2max_ in the ∼130% MAP group compared to the 105% CP group accompanied with superior improvements in VO_2max_ for the ∼130% MAP group. However, no correlation was found between T@VO_2max_ and the improvements in VO_2max_. Likewise, Rønnestad et al. (2022) measured T@VO_2max_ in elite cross-country skiers during 6 × 5 min intervals and found a tendency towards a positive relationship between T@VO_2max_ and improvements in VO_2max_ (r = 0.54, *p* = 0.071). However, the intervention group did not demonstrate larger improvements in VO_2max_ compared to the control condition. Although the evidence is scarce in this respect, there is consensus (ÅStrand et al., 1960; Wenger and Bell, 1986; Buchheit and Laursen, 2013a) that a specific training stimulus is needed to achieve a specific training goal. For instance, if the objective is to improve VO_2max_, T@VO_2max_ should be maximized (taxing the cardiovascular and aerobic enzymatic system to their maximum) to efficiently improve the aerobic system. Thus, in addition to the target intensity of 90%–100% VO_2max_ or p/vVO_2max_ or higher also a minimal interval duration is needed to maximize the training effects on endurance capacity.

In summary, the state of knowledge about effects of HIIT on the aerobic system and endurance performance in general is still highly debated in the literature and some questions remain to be clarified by randomized controlled trials (RCTs). Of note, the main focus of most above-mentioned studies was placed on the effects of VO_2max_, while other key endurance performance metrics like threshold performance, work economy, fractional utilization were neglected in this context.

## 5 Science of anaerobic HIIT–effects on performance and energy contribution

A reasonable assumption is that SIT (as defined in this review) is proposed to increase maximal sprint performance. However, there is little evidence to support this aspect (Rumpf et al., 2016). As a side note, in various studies, P_max_ derived from a Wingate or isokinetic sprint test (e.g., maximal lactate production rate test (Hauser et al., 2014)) is used as the main predictor of maximal sprint performance, which seems to be driven more by convenience than by empirical evidence (Dorel, 2018; Douglas et al., 2021; Ferguson et al., 2023). Instead, using a track running or cycling sprint over a certain distance is considered a more ecological valid metric for maximal sprint performance (Rumpf et al., 2016; Ferguson et al., 2023). Remarkably, it appears that SIT induces also comparable aerobic and metabolic adaptations like traditional endurance exercise (Sloth et al., 2013). This aspect is interesting, based on the fact that the main adenosine triphosphate (ATP) resynthesis during SIT exercise bouts would be expected to rely predominantly on anaerobic metabolism (McCartney et al., 1986) like breakdown of PCr and glycolysis, whereas traditional endurance training and most forms of aerobic HIIT predominantly rely on aerobic metabolism. PCr is the most immediate reserve for rephosphorylation of ATP and is, therefore, particularly important for repeated-sprint performance (Girard et al., 2011). Short recovery periods between sprints result in a gradual reduction in the absolute contribution of PCr to total ATP production (Girard et al., 2011). Assuming that the decrease in PCr stores during RSIT is a consequence of insufficient recovery times for PCr resynthesis, it is reasonable to hypothesize that this mechanism is the training stimulus to increase PCr stores (Verheijen, 2016). However, increases in P_max_ with RSIT (5-s all out with 55-s recovery) were not associated with increased resting muscle PCr concentrations in recreationally runners. Instead, this increase in P_max_ was associated with an increase in energy production from anaerobic glycolysis (Nevill et al., 1989). On the one hand, glycolytic activity is debated on the basis that sprint performance in trained males is positively related to a greater glycogenolytic rate (Bogdanis et al., 1995). On the other hand, it was also shown to be related to greater decrement in RSA in untrained females (Bishop et al., 2004). It is worth noting that several decades ago, the use of RSIT was not recommended for training purposes, based on the drop in power output and high muscle lactate concentrations (Billat, 2001b).

During a single short sprint, the contribution of the aerobic system to the total energy expenditure is limited typically accounting for <10% (Parolin et al., 1999; McGawley and Bishop, 2008; 2015). However, when sprints are repeated the level of aerobic ATP resynthesis progressively increases up to 40% during final repetitions of an e.g., RSIT (McGawley and Bishop, 2008; Girard et al., 2011; McGawley and Bishop, 2015) with athletes shown to reach even their VO_2max_ during latter sprints (Dupont et al., 2005; McGawley and Bishop, 2008; 2015). This effect is enhanced the shorter the recovery duration between intervals (Gaitanos et al., 1993; Kavaliauskas et al., 2015; Rogers et al., 2024), putting greater emphasis on the aerobic system for ATP resynthesis based on non-sufficient recovered PCr stores (Bogdanis et al., 1995). With longer recovery duration between intervals, mean power output during latter sprints can be maintained, due to almost full recovery of e.g., PCr resynthesis, enhanced lactate/H+ removal and greater contribution of non-oxidative metabolism (Rogers et al., 2024). These example demonstrate, that particularly RSA may also be limited by VO_2max_ and that an increase in VO_2max_ and/or VO_2_ kinetics, e.g., a faster rise in VO_2_ (Poole and Jones, 2012) may allow for greater aerobic contribution and consequently reduction in the decrement during RSIT (Girard et al., 2011; McGawley and Bishop, 2015). In this context, it was demonstrated that athletes with greater VO_2max_ demonstrated a lower decrement during RST (r = −0.45 to −0.75) (Dawson et al., 1993; Bishop and Spencer, 2004; Bishop and Edge, 2006; Brown et al., 2007; Rampinini et al., 2009). However, other studies have demonstrated poor or even positive correlations (r = −0.20–0.30) (Girard et al., 2011).

The rate of PCr resynthesis was shown to be related to the endurance level of participants (Yoshida and Watari, 1993; Bogdanis et al., 1996). PCr is used to perform the explosive actions (maximal sprints, accelerations) and the oxidative system therefore is linked to the restoration of PCr between the sprints (Verheijen, 2016). This example demonstrates that on the one hand the aerobic system can improve the anaerobic performance during e.g., RSIT. On the other hand, SIT and RSIT also constitute an adequate stimulus towards improvements of the aerobic system. Therefore, it remains to be clarified whether the increase of the anaerobic glycolytic contribution is linked to increased sprint and repeated-sprint performance or *vice versa*. The same applies for the role of aerobic fitness in relation to RSA and how RSIT is affecting both the PCr system with respect to size of the internal storage and rate of resynthesis and the aerobic system.

Iaia et al. (2008, 2009) demonstrated that replacing continuous moderate endurance training volume with SET (repeated 30-s running bouts at 90%–95% of speed achieved in a 30-s all-out run, separated by 3 min), i.e., SEPT, improved markers of aerobic and anaerobic endurance performance (30-s sprint test, Yo-Yo intermittent recovery test and supramaximal run performance) in trained runners, while 10 km run performance was maintained. Furthermore, it was shown that in non-athletes a 4 × 30-s all-out exercise separated by 2.5–4 min, i.e., SEPT, enhanced 30-s performance, VO_2max_ and various enzyme activity of anaerobic and aerobic pathways (MacDougall et al., 1998). Performance improvements have also resulted from repeated supramaximal HIIT (12 × 30 s at 175% PPO, 4.5 min recovery), i.e., SEPT (McKenna et al., 1996). Further, SEPT (8 × 20-s all-out efforts with 4.5–5 min active recovery) increased the body’s anaerobic capacity, e.g., measured by maximal accumulated oxygen deficit (Medbo and Burgers, 1990). In summary, exact recommendations of interval duration, recovery duration and recovery activity during SET cannot be provided at present, and further studies are needed. Therefore, in the current model, plausible ranges in the above-mentioned control parameters are presented.

## 6 Further perspectives

As already highlighted in the various HIIT categories above, the optimal interval:recovery ratio has not yet emerged from the literature and practice. According to Laursen and Jenkins (2002) little information is available concerning the optimal recovery duration between HIIT bouts. In general, coaches and researchers have used fixed interval:recovery ratios (i.e., 1 : 0.5, 1 : 1, 0.5 : 1) (Billat et al., 1999; Coyle, 1999; Smith et al., 1999; Stepto et al., 2001; Rozenek et al., 2007) or recovery durations based on heart rate returning to a fixed percentage (Acevedo and Goldfarb, 1989), e.g., 65%, of its maximum, particularly for traditional aerobic HIIT (Laursen et al., 2002). Another suggestion for controlling recovery duration is to suspend exercise until the athlete is no longer “out of breath” to determine that the PCr system is fully recovered (Verheijen, 2016). In addition, inconsistency persists with respect to the mode of the recovery between intervals. It was shown that with active recovery periods, the rapid component of recovery after HIIT seems to be enhanced, suggesting that the use of active recovery periods during HIIT should be preferred (Arslan et al., 2017). Ben Abderrahman et al. (2013) compared passive and active recovery in a 7-week HIIT study and the group with active recovery was able to achieve greater improvements in VO_2max_ despite or maybe based on a higher training volume. Wahl et al. (2014) concluded that acute differences were found between active and passive recovery and the long-term effects of both recovery modes seemed to differ. Hence, with respect to interval recovery duration and recovery mode there is still inconclusiveness and discrepancies. This is also based on lacking research with a special focus on systematic alterations in recovery duration and activity/passivity in well-controlled RCTs. The complexity of possible training effects of HIIT is also given based on that isolated manipulation of each single variable, e.g., interval duration, interval exercise modality, recovery duration, recovery activity, interval intensity, etc., has in most cases a direct impact on the acute metabolic, cardiorespiratory and neuromuscular response (Tschakert and Hofmann, 2013) and consequently also effects the chronic training adaptations.

In recent years, there is an ongoing discussion on adding a new dimension termed “durability” or “resilience” to the model describing the physiological determinants of endurance exercise performance, consisting of VO_2max_ and lactate threshold, which both result in the fractional utilization of VO_2max_ that can be sustained during competition, as well as work economy/efficiency, which is important in translating performance VO_2_ into speed or power (Maunder et al., 2021; Jones, 2023). Durability in the context of endurance exercise is defined as the ability to resist fatigue and maintain performance (Jones, 2023). Studies investigating the relationship between variables of durability, e.g., fresh vs fatigued critical power or time trial performance, and traditional physiological parameters, e.g., VO_2max_ or ventilatory thresholds, derived from a physiological exercise test show conflicting results (Spragg et al., 2023a; Valenzuela et al., 2023). The contributing factors what classifies an athlete as more durable compared to other athletes with similar physiological parameters, and whether psychophysiological factors, e.g., pain resistance/tolerance in elite athletes, also play a role, remains an open question (Hutchinson, 2018). It has been shown that training time below the first ventilatory threshold across a competitive season of professional cyclists may have a positive impact on measures of durability (Spragg et al., 2023b). However, to the best of our knowledge, there are hardly any studies available investigating the effect of HIIT on measures of durability. One study by Almquist et al. (2021) demonstrated that the addition of maximal 30-s “sprints” (four series of 3 × 30-s maximal effort interspersed by 4-min integrated into a low intensity session of at least 4-h; i.e., SEPT integrated into low intensity training) during a 2-week high-volume cycling training camp allowed for the maintenance of gross economy in a semi-fatigued state compared to reductions in gross economy in the non-sprint group, suggesting improved durability with SEPT. Further research is needed to investigate the effects of different HIIT types on measures of durability or resilience.

Finally, it is important to recognize that individual responses to certain training modalities must be expected in training practice. Some athletes may respond remarkably positively to certain types of HIIT, while others may not respond at all or even experience negative effects, i.e., insufficient recovery or overreaching (Stöggl and Sperlich, 2014; Casado et al., 2023; Strepp et al., 2024). A variety of predeterminants, including genetic predisposition, baseline phenotype, training status, recovery and “ready to train status”, as well as lifestyle factors such as sleep and nutrition, may contribute to individual training responses (Mann et al., 2014). In this respect, additional research is needed to gain a deeper understanding of the determinants of training response in order to achieve optimal training results. The interpretation of “response” or “non-response” can also be considered within an individual and highly depends on the variable of interest. For example, an athlete may experience an improvement in VO_2max_ (i.e., response) through aerobic HIIT wiht long intervals, while e.g., threshold performance remains unchanged (i.e., non-response) (Mann et al., 2014).

## 7 Conclusion

In the current review, we present a HIIT categorization model based on previous literature, the idea of a training goal-oriented approach and an attempt towards decomplexation with regards to guidelines for interval intensities, interval durations and recovery durations. The objective was to find a consensus between different definitions from the existing literature, introduce clear definitions, obtain a stringent terminology, consider all in literature proposed HIIT categories and to establish a prerequisite for future analyses. The proposed types of HIIT and their expected training effects on various metrics of endurance, sprint and repeated-sprint performance still bare various open questions. A systematic review with meta-analysis is warranted to strengthen and fine-tune the proposed HIIT “toolbox” and to provide more clarity in the above highlighted questions and discrepancies.
